# Supplementary material for: Dynamics of Complex Systems Built as Coupled Physical, Communication and Decision Layers
Source: PLoS One. 2016 Jan 5;11(1):e0145135. doi: 10.1371/journal.pone.0145135 (PMC4701467; doi:10.1371/journal.pone.0145135)
Supplement: S1 File — (PDF) [file pone.0145135.s001.pdf]

Supporting Information: Understanding the feedback of the physical layer on the agents behavior

To better understand mid-size systems, we can imagine that two agents cooperating for a long time will eventually end up with only one resistor each. If nobody else is cooperating, they will not receive any positive gain, being trapped in the cooperative state. Eventually, other agents will reach their tipping point, resulting in a change of their behavior. When this happens, agent i, which is trapped at time-step t, experiences $\Delta a_i[t] = 0$, $\Delta r_i[t] < 0$ and $a_{\text{avg}}[t] \approx \mu$. We need then to look for a gain bigger than $\lambda_{\min}$ to free the trapped agent i

$$\lambda_i[t] = -\frac{1}{N\mu}\Delta r_i[t] > \lambda_{\min},$$

where this can only be true for systems smaller than

$$N < -\frac{\Delta r_i[t]}{\lambda_{\min}\mu}.$$

For instance, the previous used values $\lambda_{\min} = 0,0005$, $\mu = 100$ and $\Delta r_i[t] = -1$ lead to $N < 20$. This means that, in systems larger than twenty, the system needs to have a variation of $|\Delta r_i[t]| > 2$. In other words, if almost all agents are trapped in cooperative solidarity, the other agents, which are still increasing or reducing the number of resistors, have to synchronize in a way that the overall change is more than two resistors for every time step.

From a different perspective, we can see that the system needs more than fifty agents to synchronously reducing their resistors to free the trapped agents when $N = 1000$ since

$$-\Delta r_i > N\mu\lambda_{\min}.$$

This event, however, is very unlike and explains the reason of cooperation being dominant in large systems; almost all agents are trapped in solidarity cooperation and will not see a sufficient gain to stop their solidarity. It furthermore explains why we see so much cooperation for high values of $\lambda_{\min}$ in Fig 5, the feedback can never be big enough to release the trapped agents. So the only way for them to be freed is through a random communication error.

We can now also understand the behavior of mid-size systems. Once the system pass its optimal point, more and more agents become trapped in solidarity cooperation. At a certain point, however, the agents that are still gaining power (mostly due to the fact that the other agents left so many resources available for using) come close enough to the optimal point and start to cooperate as well. Since they have added a lot of resistors, it will take them some time-steps to reach the lower bound (i.e. one resistor). It is also very likely that there will be a sufficient number of agents synchronizing so that the trapped ones can experience gains to trigger a change in their state. This will lead a new wave of defecting until the resource is once again overused, generating then the wave-like system behavior. It is also worth noting that the length of the “wave-fronts” is not predictable due to the need for synchronization to trigger a global change.

We have not yet explained the difference in behavior that is visible in Fig 6 when small systems are considered. Different from the their bigger systems, the smaller ones seem to stay much closer to the optimal point, while they also share the power a fairer ways. These facts can be explained because, for system below a certain size, the term in equation (3) that provides individual feedback is

$$-\frac{2}{N}\frac{\Delta a_i[t]}{a_{\text{avg}[t]} + \mu} > \lambda_{\min},$$

which is bigger than the minimal gain.

With the same values as above and $\Delta a_i[t] = 1$, the inequality is only true for systems with $N < 10$. If an agent has too many resistors, it will mostly experience a positive gain by cooperating and also by changing its behavior before it is trapped in solidarity. This makes the system much more efficient and fair.

Another question that can be posed is about the possibility of setting the parameters in such a way that the wave-like behavior we see in mid-size system can be seen in small or in large systems. Surprisingly, it turns out that this is not possible. To create similar conditions to mid-sized systems in a big system, we need to lower the speed of reduction needed by the system to free trapped agents such that

$$-\Delta r_i[t] > N\mu\lambda_{\min},$$

We could then lower either μ or $\lambda_{\min}$. However a decrease of $\lambda_{\min}$ would mean an increase in $a_{\max}$ so that agents are much more likely to add more resistors and less likely to become cooperative. Similarly, a decrease of μ will decrease the optimal number of resistors in the system

$$n^* = N\mu,$$

so that when the agents reach their natural limit $a_{\max}$ they are far the optimal point of the system. Both conditions will lead to a state where the agents will overuse the system up to a point that is far away from the optimum without ever recovering.

For small systems, we would need to raise either μ or $\lambda_{\min}$. When $N < 20$, it is unlike that a big fraction of the system get trapped while the rest synchronizes in a way to release them. Then, wave-like behavior cannot be observed in small-size systems.
